# MicroRNA-203 inhibits epithelial-mesenchymal transition, migration, and invasion of renal cell carcinoma cells via the inactivation of the PI3K/AKT signaling pathway by inhibiting CAV1

**DOI:** 10.1080/19336918.2020.1827665

**Published:** 2020-11-22

**Authors:** Ning Han, Hai Li, Hui Wang

**Affiliations:** a Department of Radiology, China-Japan Union Hospital of Jilin University, Changchun, P. R. China; b Department of Urology Surgery, China-Japan Union Hospital of Jilin University, Changchun, P. R. China; c Department of Ultrasound, China-Japan Union Hospital of Jilin University, Changchun, P. R. China

**Keywords:** MicroRNA-203, CAV1, PI3K/AKT signaling, renal cell carcinoma, proliferation, apoptosis, epithelial-mesenchymal transformation, migration, invasion

## Abstract

The present study aimed to evaluate the underlying mechanism of microRNA-203 (miR-203) in renal cell carcinoma (RCC) involving the PI3K/AKT signaling pathway. The results revealed downregulated miR-203 and upregulated CAV1 in RCC tissues. Upregulated miR-203 and downregulated CAV1 increased E-cadherin expression and cell apoptosis, decreased β-catenin and N-cadherin expression and cell proliferation, migration and invasion, and blocked cell cycle entry. CAV1, a target gene of miR-203, decreased by up-regulated miR-203, and silencing CAV1 led to the inactivation of PI3K/AKT signaling pathway. In conclusion, our findings suggested that miR-203-mediated direct suppression of CAV1 inhibits EMT of RCC cells via inactivation of the PI3K/AKT signaling pathway.

## Introduction

Renal cell carcinoma (RCC), the malignant tumors consisting of renal parenchyma and renal pelvis [1], accounts for 90% of the renal malignancies [2]. Its incidence rate varies with territories and genders; for example, males are approximately twice more likely to suffer from RCC than females [3]. In addition, RCC incidence has also been linked to the following factors: smoking [4], obesity [5], hypertension, and antihypertensive treatment [6]. In terms of therapeutic approaches, RCC, with multiple drug resistance genes, is characterized by inherent resistance to chemotherapy, which makes it one of the most difficult malignant tumors to treat [7]. Therefore, the prognosis of patients with metastatic RCC (mRCC) is so dismal that the 5-year survival rate is less than 10% [8]. Hence, it is imperative to study the molecular basis of RCC for the design of novel therapeutics that helps increase survival rates.

Accumulating evidence has reported that many microRNAs (miRs), such as miR-646 [9], miR-184 [10], and miR-144 [11] play a pivotal role in RCC tumorigenesis. Located at chromosome 14q32-33, miR-203 is reported to be a tumor suppressor in chronic myeloid leukemia (CML) [12]. In addition, it has been reported that decreased miR-203 expression led to the enhancement of RCC cell growth and metastasis via FGF2 overexpression [13]. Furthermore, it is predicted that miR-203 can bind with the 3ʹ-untranslated region (UTR) region of caveolin-1 (CAV1) gene in the biological prediction site. Previous finding demonstrated that CAV1 is an important structural protein in the caveolae and it cooperates with the AKT/mTOR signaling pathway in advanced RCC [14]. In addition, former studies have found that there is a strong association between the P13 K/AKT/mTOR signaling pathway and the occurrence of RCC, due to its ability to accelerate cell cycle, reduce apoptosis, and promote the migration of tumor cells [15,16]. The aforementioned data led to the hypothesis that miR-203 functions as a tumor suppressor in RCC by modulating the PI3K/AKT signaling pathway via CAV1. This study evaluated the mechanism of miR-203 in EMT, proliferation, migration, invasion, and apoptosis of RCC cells via the PI3K/AKT signaling pathway by targeting CAV1, which further elucidated the molecular mechanism of RCC progression, and might provide new findings for targeted therapy.

## Results

### CAV1 is upregulated in RCC through microarray profiling

**Figure 1.**
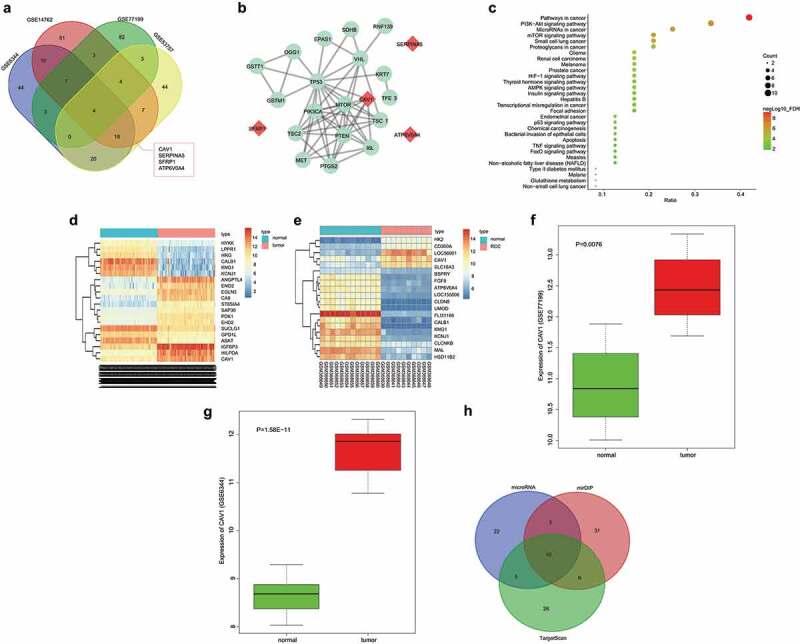
Bioinformatics predicts that CAV1 is upregulated in RCC

Notes: Panel a, the first 100 differential genes from gene datasets GSE53757, GSE14762, GSE77199 and GSE6344 reveals that there are 4 genes that are differentially expressed in all 4 gene datasets; Panel b, the network of interactions between the RCC differential genes and the known diseases genes, among which the red represents the differential genes, and green represents the known diseases genes; Panel c, KEGG enrichment results of the RCC differential genes and known disease genes; Panel d and e, expression heatmaps of the top 20 differential genes in gene datasets GSE53737 and GSE14762 respectively. The horizontal coordinate represents the sample numbers while the ordinate indicates the differential genes. The histogram on the upper right is the color gradation, and each rectangle in the figure corresponds to a sample expression value. Red indicates high expression, and blue indicates low expression; Panel f and g, CAV1 expression in gene datasets GSE77199 and GSE6344 respectively; Panel h, microRNA, TargetScan and mirDIP prediction can control the miRNA results of CAV1 by comparing the top 50 miRNAs, and there are 10 miRNAs.

In order to select mRNA related to cell proliferation, migration, invasion, apoptosis, and EMT of RCC, bioinformatics prediction was performed. Based on the gene datasets (GSE53757, GSE14762, GSE77199, and GSE6344) of kidney cancer in GEO database and according to the conditions of *P.Value* < 0.05 and |LogFoldChange| >2.0, the differentially expressed genes (DEGs) were selected, and the top 100 genes of the 4 gene datasets were selected to compare with each other, and Venn diagram (Figure 1(a)) was generated. The Venn diagram revealed 4 intersecting genes (CAV1, SERPINA5, SFRP1, and ATP6V0A4). RCC-related genes were retrieved using DisGeNET, and the top 20 ones were selected as known disease genes. Gene interaction was carried out between pre-selected intersecting DEGs and known disease genes, and gene interaction network (Figure 1(b)) was generated. In this network, a single differential gene, CAV1 was found to be associated with disease genes, and therefore the CAV1 gene expression differences may be of great significance in the RCC progression. The Kyoto Encyclopedia of Genes and Genomes (KEGG) signaling pathway enrichment results of RCC differential genes and disease genes are shown in Figure 1(c). These five RCC-related genes TP53, PIK3CA, IL6, TSC2, and PTEN were not only associated with CAV1, but also were concentrated in the PI3K/AKT signaling pathway, suggesting that CAV1 could potentially affect RCC through the PI3K/AKT signaling pathway. We plotted the expression heatmaps of the top 20 DEGs in GSE53757 (Figure 1(d)) and GSE14762 (Figure 1(e)): compared with normal renal tissues, RCC tissues presented with increased CAV1 expression. CAV1 was highly expressed in cancer tissues in gene dataset GSE77199 (Figure 1(f)) and gene dataset GSE6344 (Figure 1(g)). Among miRNAs that were predicted in microRNA, TargetScan and mirDIP to be able to regulate CAV1, the top 50 genes from each prediction result were selected to compare each other. There were 10 intersecting miRNAs (Figure 1(h)), and the information of miRNA-CAV1 in microRNA database are shown in Table 1. The lower the mirSVR score was, the stronger the combination stability of miRNA-mRNA and the higher the probability of the miRNA as the corresponding down-regulated gene would be [17]. The lowest value of mirSVR score was hsa-miR-203a-3p, and studies have shown a low expression of miR-203 in RCC [13]. The underlying molecular mechanism remains unclear, but we hypothesized that miR-203 could target CAV1 to mediate PI3K/AKT signaling pathway in RCC.

**Table 1.** Prediction information of miRNA targeting CAV1

miRNA	mirSVR score	PhastCons score
hsa-miR-203a-3p	−0.8746	0.6579
hsa-miR-199a-5p	−0.8069	0.7279
hsa-miR-199b-5p	−0.8069	0.7279
hsa-miR-1271-5p	−0.547	0.573
hsa-miR-96-5p	−0.5399	0.573
hsa-miR-506-3p	−0.5028	0.6214
hsa-miR-133b	−0.4605	0.5918
hsa-miR-7-5p	−0.4472	0.5729
hsa-miR-192-5p	−0.152	0.6086
hsa-miR-338-3p	−0.1487	0.6665

Notes: miR, microRNA.

### RCC correlates to CAV1 overexpression

**Figure 2.**
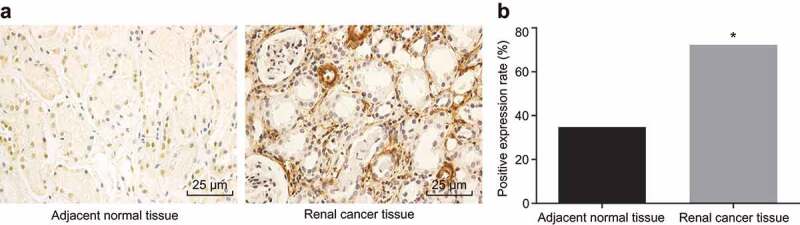
IHC of cancer and adjacent normal tissues shows that CAV1 is overexpressed in RCC tissues

Notes: Panel a, The positive-CAV1 cells shows linear yellow-brown cytomembranes in adjacent normal tissues and RCC tissues (from female patients at stage Ⅱ) (× 400); Panel b, statistics of CAV1-positive cells in cancer and adjacent normal RCC tissues; n = 112. IHC, immunohistochemistry; RCC, renal cell carcinoma; CAV1, caveolin-1; *, *p* < 0.05, compared with the adjacent normal tissues.

IHC was performed to measure positive expression rate of CAV1 in RCC tissues and adjacent normal tissues to explore effect of CAV1 on RCC. As demonstrated in Figure 2(a,b), the positively stained CAV1 cells were observed to have linear yellow-brown cytomembranes. Adjacent normal tissues exhibited evenly distributed and regularly arranged blood vessels, while vessels in RCC tissues presented with an uneven distribution and random arrangement. Some of the RCC micro-vessels even anastomosed into meshes and lost the vascular lumen structure. Compared with the adjacent normal tissues, the positive rate of CAV1 in the RCC tissue was significantly increased (*p*< 0.05).

### MiR-203 is downregulated but CAV1 is reciprocal in RCC tissues

**Figure 3.**
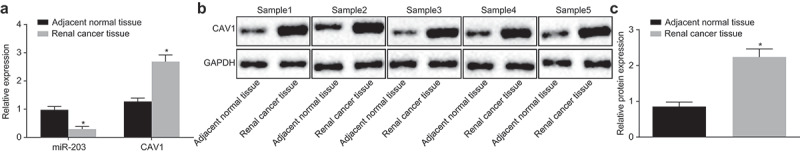
MiR-203 is downregulated while CAV1 is upregulated in RCC tissues

Notes: Panel a, the determination by RT-qPCR demonstrates that the RCC tissues exhibit lower miR-203 expression and higher mRNA level of CAV1 than adjacent normal tissues (sample 1 from male patients at stage II, sample 2 from female patients at stage III, sample 3 from male patients at stage II, sample 4 from female patients at stage III, and sample 5 from male patients at stage III); Panel b and c, the determination by western blot analysis demonstrates that the RCC tissues exhibit higher protein level of CAV1 than adjacent normal tissues; n = 112; RT-qPCR, reverse transcription quantitative polymerase chain reaction; RCC, renal cell carcinoma; CAV1, caveolin-1; miR-203, microRNA-203; *, *p* < 0.05, compared with adjacent normal tissues.

RT-qPCR and western blot analysis was conducted to determine the expression of miR-203 and CAV1 in RCC tissues and adjacent normal tissues. The results (Figure 3(a-c)) illustrated that miR-203 expression was significantly lower in RCC tissues than in adjacent normal tissues, while CAV1 mRNA and protein level were remarkably higher (*p*< 0.05). These findings provided evidence that RCC tissues had decreased miR-203 expression and increased mRNA and protein level of CAV1.

### Low expression of miR-203 and overexpression of CAV1 expression contributes to the development of RCC

RT-qPCR was used to determine the expression of miR-203 and CAV1 in RCC and adjacent normal tissues. Compared with adjacent normal tissues, the expression of miR-203 in RCC tissues showed a significant decline (*p*< 0.05). Combined with the analysis of clinicopathological parameters, the expression of miR-203 and CAV1 in patients suffering from RCC was closely correlated with tumor-node-metastasis staging and degree of tumor differentiation, while there were no links with age, gender and tumor size. The expression of miR-203 in patients with lower degree of differentiation, or III/IV stage was significantly lower than that in patients with higher degree of differentiation, or I/II stage, respectively, whereas CAV1 expression exhibited opposite trends (Table 2). Hence, the results indicated that the expression of miR-203 and CAV1 was closely related to the clinical stage, and tumor differentiation degree.

**Table 2.** Correlation of miR-203 and CAV1 with RCC progression

Clinicalpathologic features	n	miR-203	*p*	CAV1 positive expression rate (%)	*p*
Age					
< 50	54	0.30 ± 0.09	0.243	40 (74.07)	0.689
> 50	58	0.28 ± 0.09		41 (70.69)	
tumor diameter (cm)					
< 4	47	0.30 ± 0.07	0.557	33 (70.21)	0.672
> 4	65	0.29 ± 0.10		48 (73.85)	
Gender					
male	68	0.29 ± 0.09	0.253	48 (70.59)	0.610
female	44	0.31 ± 0.09		33 (75.00)	
TNM Staging					
I,II	58	0.35 ± 0.07	< 0.001	32 (55.17)	< 0.001
III,IV	54	0.24 ± 0.08		49 (90.74)	
Tumor differentiation degree					
High + Moderate	70	0.31 ± 0.08	0.004	44 (62.86)	0.004
Poor + Non	42	0.26 ± 0.08		37 (88.10)	

Notes: CAV1, caveolin 1; TNM, tumor node metastasis; LNM, lymph node metastasis; miR-203, microRNA-203.

### CAV1 is a target gene of miR-203

**Figure 4.**
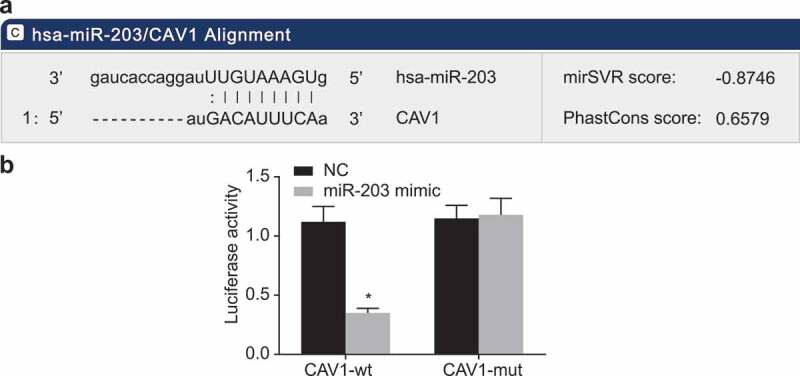
MiR-203 directly targets CAV1

Notes: Panel a, miR-203 is presumed to binding to the 3ʹuntranslated regions (3ʹUTR) of CAV1 in microRNA.org; Panel b, a combination of CAV1-wt and miR-203 mimic significantly reduces the luciferase activity while a combination of CAV1-mut and miR-203 mimic does not change the luciferase activity, suggesting miR-203 binds to the 3ʹUTR of CAV1; CAV1, caveolin-1; miR-203, microRNA-203; NC, negative control; *, *p* < 0.05, compared with the NC group. The experiment was repeated 3 times independently.

The bioinformatics prediction website (microRNA.org) was used for the target gene analysis of miR-203, and dual-luciferase reporter assay was also conducted to confirm whether CAV1 was the direct target gene of miR-203. The results (Figure 4(a)) showed that there was a specific binding region between the CAV1 gene sequence and the miR-203 sequence, which confirmed that CAV1 was the target gene of miR-203. The results of dual-luciferase reporter assay confirmed that CAV1 was the target of miR-203. The results showed that compared with the negative control (NC) group, the luciferase activity of wt co-transfected (miR-203 mimic/wt-CAV1) group in the miR-203 mimic group decreased (*p* < 0.05); whereas the luciferase activity of mutant 3ʹUTR showed no significant difference (*p*> 0.05; Figure 4(b)). All of the aforementioned findings suggested that miR-203 could specifically bind to CAV1 and CAV1 was the direct target gene of miR-203.

### 786-O cell line is selected for the subsequent study

**Figure 5.**
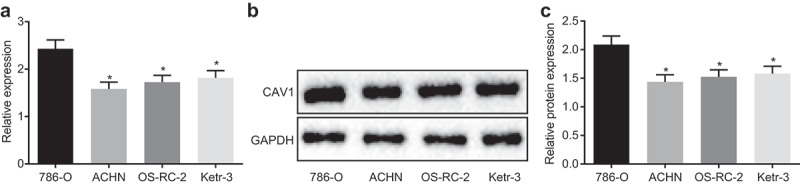
786-O cell line is suitable for the subsequent experiments

Notes: Panel a, the determination by RT-qPCR demonstrates that 786-O cell line exhibits the highest mRNA level of CAV1 than other cell lines ACHN, OS-RC-2 and ketr-3ACHN; Panel b and c, the determination by western blot analysis demonstrates that 786-O cell line exhibits the highest protein level of CAV1 than other cell lines ACHN, OS-RC-2 and ketr-3ACHN; RT-qPCR, reverse transcription quantitative polymerase chain reaction; CAV1, caveolin-1; *, *p* < 0.05 compared with 786-O cells. The experiment was repeated 3 times independently.

RT-qPCR and western blot analysis were used to screen the cell line with the highest expression of CAV1 among human renal clear-cell adenocarcinoma cell lines 786-O, ACHN, OS-RC-2, and ketr-3. The results of RT-qPCR (Figure 5(a-c)) showed that the expression of CAV1 mRNA was the highest in 786-O cell line among the remaining three (ACHN, OS- and ketr-3). Meanwhile, western blot analysis also revealed that in comparison with other cell lines, CAV1 protein expression in 786-O cell line was significantly higher. Hence, 786-O cell line was selected for subsequent experiments.

### MiR-203 inhibits CAV1 and the PI3K/AKT signaling pathway in RCC cells

**Figure 6.**
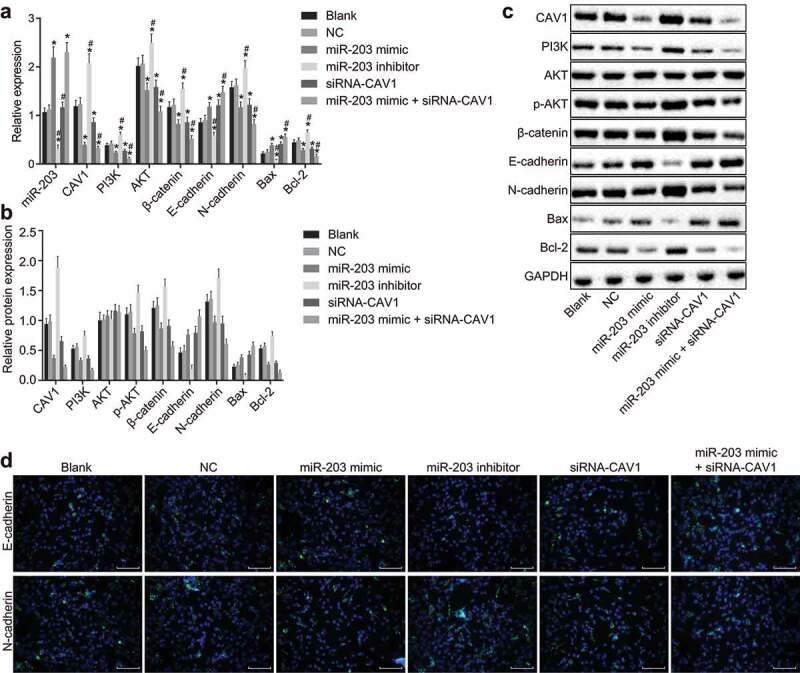
MiR-203 suppresses functional CAV1 and inactivates PI3K/AKT signaling pathway in RCC cells

Notes: Panel a, the determination by RT-qPCR demonstrates that ectopic expression of miR-203 decreases the mRNA levels of CAV1, PI3K, AKT, β-catenin, N-cadherin and bcl-2, and increases the mRNA levels of E-cadherin and Bax; Panel b and c, the determination by western blot analysis demonstrates that ectopic expression of miR-203 decreases the protein levels of CAV1, PI3K, AKT, phosphorylated AKT, β-catenin, N-cadherin and bcl-2, and increases the protein levels of E-cadherin and Bax; Panel d, the expression of EMT-relate factors (E-cadherin and N-cadherin) detected by immunofluorescence (× 200); RT-qPCR, Reverse transcription quantitative polymerase chain reaction; CAV1, caveolin-1; miR-203, microRNA-20**3**; PI3K, phosphoinositide 3-OH kinase; Bax, bcl-2 associated x protein; bcl-2, B-cell lymphoma-2; *, *p* < 0.05, compared with the blank group; ^#^, *p* < 0.05, compared with miR-203 mimic group; Blank: no sequence was transfected; the experiment was repeated 3 times.

To explore the mechanism among miR-203, CAV1, and the PI3K/AKT signaling pathway in RCC, RT-qPCR and western blot analysis were conducted to detect miR-203, CAV1, PI3K, AKT, β-catenin, N-cadherin, bcl-2, E-cadherin, and Bax expression. RT-qPCR and western blot analysis (Figure 6(a-c)) showed that there was no significant difference in gene expression between the blank group and the NC group (all *p*> 0.05). Compared with the blank group and the NC group, the expression of miR-203 were remarkably increased in the miR-203 mimic group and the miR-203 mimic + siRNA-CAV1 group (all *p* < 0.05), decreased in the miR-203 inhibitor group (all *p* < 0.05), and remained nearly unaffected in the siRNA-CAV1 group (*p*> 0.05). In the miR-203 inhibitor group, the mRNA and protein expression of CAV1, PI3K, phosphorylated AKT, β-catenin, N-cadherin, and bcl-2 were all significantly increased (all *p* < 0.05) while expression of E-cadherin and Bax showed a notable decrease (all *p* < 0.05). However, the miR-203 mimic, siRNA-CAV1, and miR-203 mimic + siRNA-CAV1 groups presented with the opposite results (all *p* < 0.05). The comparison between the miR-203 mimic group and the siRNA-CAV1 group showed no significant differences in the expression of each gene (*p*> 0.05). The mRNA and protein level of E-cadherin and Bax were significantly higher (all *p* < 0.05), while those expression of CAV1, PI3K, phosphorylated AKT, β-catenin, N-cadherin, and bcl-2 were significantly lower in the miR-203 mimic + siRNA-CAV1 group than in the miR-203 mimic group (all *p* < 0.05). Furthermore, the expression of E-cadherin and N-cadherin, the related factors of EMT, was detected by immunofluorescence, and the findings revealed the same trend as mentioned above (Figure 6(d)). These results indicated that the up-regulation of miR-203 could suppress CAV1 and the PI3K/AKT signaling pathway.

### MiR-203 inhibits cell proliferation via CAV1 in RCC cells

**Figure 7.**
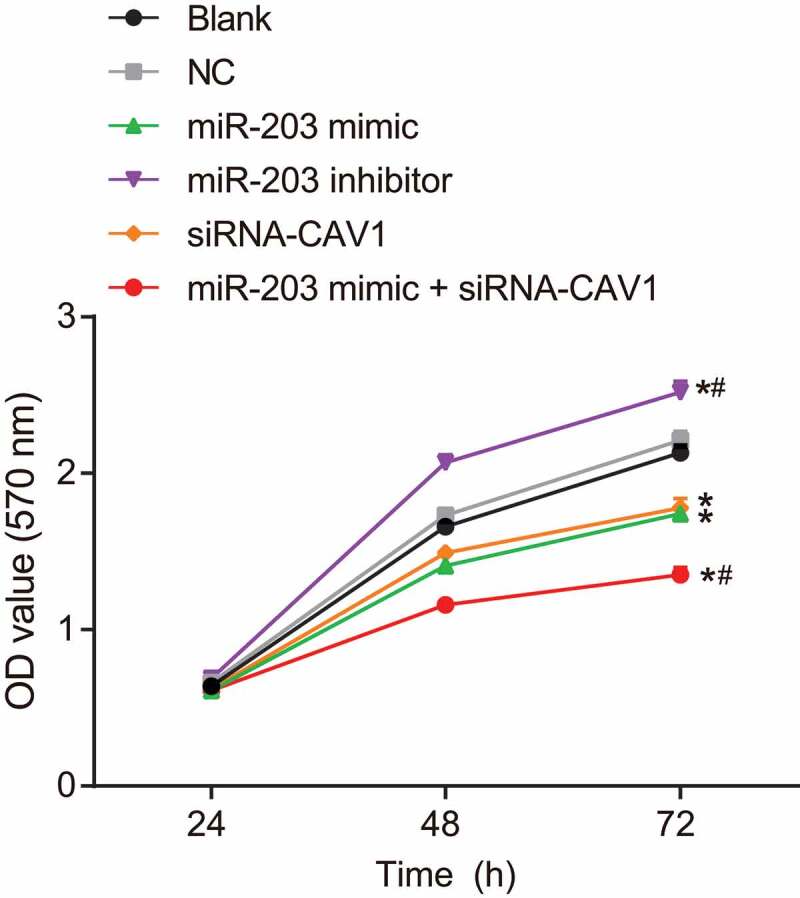
MTT showed that miR-203 inhibited cell proliferation via CAV1 in RCC cells

Notes: The experiment was repeated 3 times; RCC, Renal cell carcinoma; MTT, 3-(4,5)-dimethylthiahiazo(-z-y1)-3,5-di-phenytetrazoliumromide; miR-203, microRNA-20**3**; CAV1, caveolin-1; *, *p* < 0.05, compared with blank group; ^#^, *p* < 0.05, compared with miR-203 mimic group.

MTT assay was performed to investigate whether miR-203 affects RCC cell proliferation. MTT test results (Figure 7) demonstrated that after 24 h of transfection, the viability of each group showed no significant difference, while there was a significant difference in viability between the groups after 48 h and 72 h of transfection. There was no significant difference in cell viability between the blank group and the NC group at each time point, while cell viability was markedly increased at 48 h and 72 h in the miR-203 inhibitor group compared with the blank group and the NC group (*p* < 0.05). As for the remaining three groups (the miR-203 mimic group, the siRNA-CAV1 group and the miR-203 mimic + siRNA-CAV1 group), cell viability was significantly decreased at 48 h and 72 h (*p* < 0.05). Taken together, these results indicate that miR-203 inhibits RCC cell proliferation by targeting CAV1.

### MiR-203 inhibits cell migration via CAV1 in RCC cells

**Figure 8.**
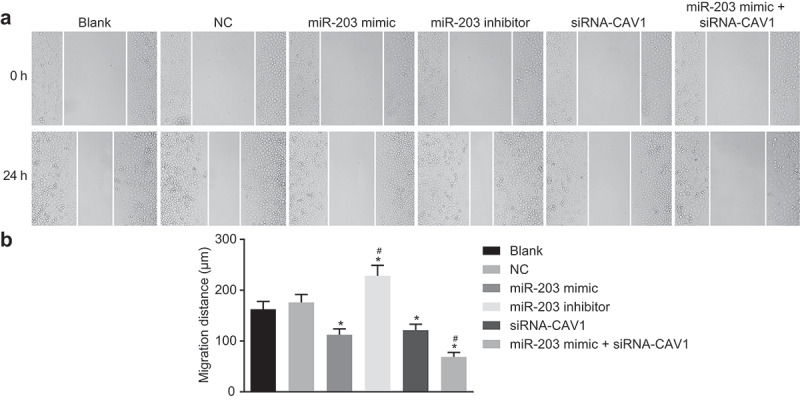
miR-203 inhibits cell migration via CAV1 in RCC cells determined by scratch test

Notes: Panel a, under the inverted microscope, the wound closure becomes faster 24 hours after miR-203 downregulation (× 200); Panel b, cell migration distance is indicated in a representative histogram;*, *p* < 0.05, compared with the blank group; ^#^, *p* < 0.05, compared with the miR-203 mimic group; RCC, renal cell carcinoma; miR-203, microRNA-203; CAV1, caveolin-1. The experiment was repeated 3 times.

Scratch test was conducted to measure cell migration. The test results at 0 h and 24 h (Figure 8(a,b)) showed no significant difference in migration ability between the blank group and the NC group (*p*> 0.05). Compared with the bank group and the NC group, cell migration in miR-203 inhibitor group were dramatically increased (*p* < 0.05) and showed a sharp decrease in the miR-203 mimic group, the siRNA-CAV1 group, and the miR-203 mimic + siRNA-CAV1 group (*p* < 0.05). Compared with the miR-203 mimic group, the migration of the miR-203 mimic + siRNA-CAV1 group was significantly reduced (*p* < 0.05). These findings revealed that miR-203 inhibits RCC cell migration through the inhibition of CAV1.

### CAV1 silencing is responsible for the inhibitory effect of miR-203 on cell invasion in RCC cells

**Figure 9.**
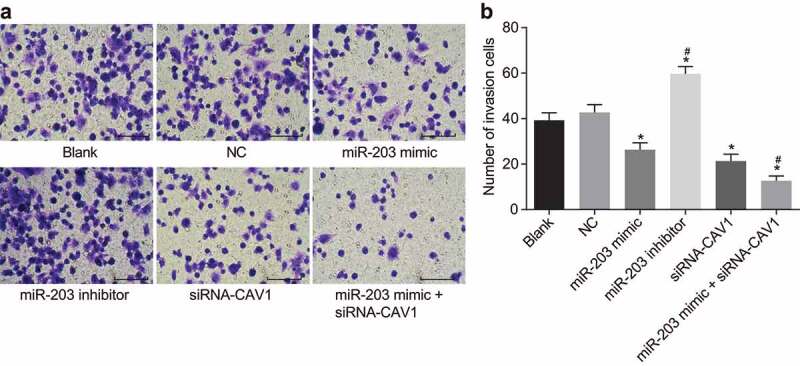
Transwell assay reveals that miR-203 inhibits cell invasion in RCC cells

Notes: Panel a, under the inverted microscope, the cells in the basolateral chamber are increased after miR-203 downregulation (× 200); Panel b, the number of cells passing through the Matrigel from apical chamber to basolateral chamber is indicated in a representative histogram; RCC, renal cell carcinoma; miR-203, microRNA-203; CAV1, caveolin-1; *, *p* < 0.05, compared with the blank group; ^#^, *p* < 0.05, compared with the miR-203 mimic group. The experiment was repeated 3 times.

Transwell assay was used as a reliable method to detect cell invasion. The results (Figure 9(a,b)) showed that there was no significant difference in the number of cells transferred from the upper chamber to the lower chamber between the blank group and the NC group (*p*> 0.05). Compared with the blank and NC groups, the number of transferring cells in the miR-203 inhibitor group was significantly increased (*p* < 0.05), but sharply decreased in the miR-203, siRNA-CAV1 and miR-203 mimic + siRNA-CAV1 groups (*p* < 0.05). Compared with the miR-203 mimic group, the number of cells transferred from the upper chamber to the lower chamber in the miR-203 mimic + siRNA-CAV1 group was significantly decreased (*p* < 0.05). These findings suggested that miR-203 represses cell invasion in RCC cells via CAV1.

### MiR-203 blocks cell cycle entry and enhances cell apoptosis through CAV1 in RCC cells

**Figure 10.**
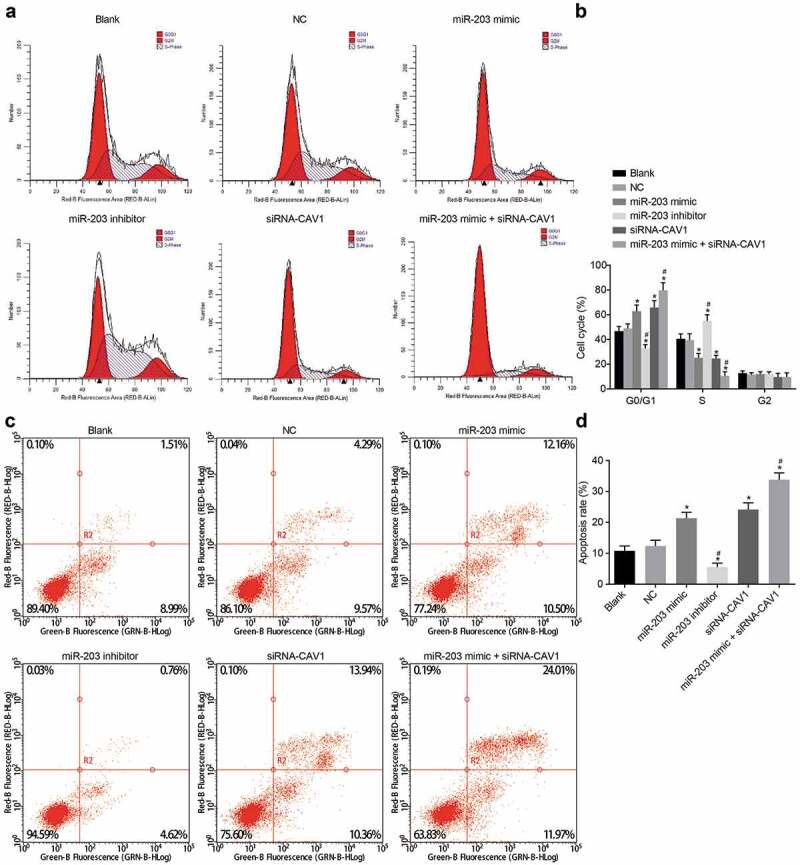
The flow cytometry analysis indicates that miR-203 blocks cell cycle entry and promotes cell apoptosis in RCC cells through CAV1-dependent PI3K/AKT signaling pathway

Notes: Upregulated expression of miR-203 induces cell cycle arrest at the G1 phase; inhibitor-mediated knockdown of miR-203 induces cell cycle arrest at the S phase. Panel a, the DNA content of PI-stained cells at the G0/G1, S, and G2 phases; Panel b, the percentage of PI-stained cells at the G0/G1, S, and G2 phases is indicated in a representative histogram; Panel c, cells in the scatter plots in which the upper left quadrant identifies the necrotic cells (annexin V-/PI+), the upper right quadrant identifies the late apoptotic cells (annexin V+/PI+), the lower left quadrant identifies the live cells (annexin V-/PI-), and the lower right quadrant identifies the early apoptotic cells (annexin V+/PI-); Panel d, the percentage of early and late apoptotic cells is indicated in a representative histogram; RCC, renal cell carcinoma; miR-203, microRNA-203; CAV1, caveolin-1; *, *p* < 0.05, compared with the blank group; ^#^, *p* < 0.05, compared with the miR-203 mimic group. The experiment was repeated 3 times.

Flow cytometry was performed to assess cell cycle distribution and apoptosis of RCC cells under the influence of miR-203. The results of flow cytometry (Figure 10(a-d)) showed no significant difference in cell cycle and apoptosis rate between the blank group and the NC group (*p*> 0.05). Compared with the blank group and the NC group, the proportion of cells in phase G1 in the miR-203 mimic, siRNA-CAV1 and miR-203 mimic + siRNA-CAV1 groups was significantly increased while the proportion of cells in phase S was significantly decreased, indicating that the apoptosis rate of cells was significantly increased (all *p* < 0.05). As for the miR-203 inhibitor group, when compared with the blank and the NC groups, more cells were arrested in phase S and fewer in phase G1, indicating a notable decline in cell apoptosis (all *p* < 0.05). In comparison with the miR-203 mimic group, the miR-203 mimic + siRNA-CAV1 group had more cells arrested in phase G1 and fewer in phase S, demonstrating a significant rise in cell apoptosis (*p* < 0.05). These findings suggested that up-regulated miR-203 could inhibit cell cycle and promote cell apoptosis in RCC cells by targeting CAV1.

## Discussion

Previous studies have shown that miRNAs have the function of ‘tumor suppressor genes’ or ‘oncogenes’ in the process of tumor development, and regulate multiple cell processes in RCC progression [18,19]. The tumor suppressive role of miR-203 in RCC has been investigated in recent studies, revealing a wide range of molecular mechanisms of miR-203 involved in the regulation of RCC cells. For example, Mingxi Xu *et al*. demonstrated that decreased miR-203 expression enhanced RCC cell growth and metastasis via FGF2 overexpression [13]. However, considering many potential targets governed by each miRNA, one challenge in understanding functional miRNA contributions in cancer cell behaviors depends on the identification of bona fide molecular targets. Hence, in this study, we clarified the mechanism by which biological function of miR-203 affects RCC cell. Furthermore, we found that miR-203 blocked PI3K/AKT signaling pathway by inhibiting CAV1, thus repressing EMT, migration, and invasion of RCC cells.

Initially, miR-203 was confirmed to be significantly downregulated in RCC and its overexpression inhibited RCC proliferation, invasion, and migration and promoted cell apoptosis. As described in a previous study, the silencing of miR-203 can lead to upregulation of snail homolog 2 [20], and that the snail was overexpressed in advanced RCC [21]. Moreover, the inhibitory effect on the malignant phenotypes of RCC exerted by enforced miR-203 expression was approved by negatively targeting FGF2 [13]. Furthermore, a previous study has already demonstrated that the up-regulation of miR-203 contributes to the suppression of cancer progression and metastasis, and is implicated in the inhibition of cell proliferation, invasion, migration, and EMT in multiple cancer cells including tongue squamous cancer and bladder cancer [22]. Therefore, we hypothesized that the overexpression of miR-203 can promote the apoptosis of RCC cells and inhibit their proliferation, migration, and invasion. In the following experiments, we demonstrated that miR-203 exerted its functional role on RCC cells by targeting the CAV1 via the PI3K/AKT signaling pathway.

In the following experiments, RCC cells were found to have overexpressed CAV1, and exposure to miR-203 mimic resulted in an increased gene expression of E-cadherin as well as reduced levels of CAV1, PI3K, AKT, β-catenin, and N-cadherin. The downregulation of CAV1 expression by siRNA led to the inhibition of RCC cell proliferation, migration, and invasion, while apoptosis was promoted. Based on the findings from online analysis software, our study identified CAV1 as a putative target gene of miR-203. CAV1, a principal structural component of caveolar membrane domains, plays a significant role in tumorigenesis [23]. For example, Liang *et al*. considered CAV1 as an oncogene in bladder cancer as it promotes invasive phenotypes by inducing EMT through Slug overexpression, and the whole process occurs through the activation of PI3K/AKT signaling pathway [24] and Henriett Butz *et al*. found that CAV1 protein was upregulated in ccRCC compared to its normal counterparts and it was associated to patient survival [25]. Another study also revealed that CAV1 was overexpressed in clear cell RCC [26]. In addition, Han *et al*. found that in paclitaxel-resistant lung cancer A549/Taxol cells, the knockdown of CAV1 significantly inhibited cell proliferation and induced cell apoptosis via the inhibition of AKT [27]. In addition, accumulating evidence demonstrated that reduced CAV1 inhibited cell proliferation and promoted apoptosis by blocking the PI3K/AKT signaling pathway [23,28]. In line with our findings, a study conducted by Zhao *et al*. reported that growth and metastasis of RCC were inhibited through the suppression of the PI3K/AKT signaling pathway [29]. Moreover, we found that miR-203 inhibited cell apoptosis through its indirect effects on anti-apoptotic genes including bcl-2 and pro-apoptotic genes, such as Bax. It can lead to ratio imbalance of Bax/bcl-2 and induce cell apoptosis caused by apoptotic protease activation. A key finding in a previous study confirmed that miR-203 increased pro-apoptotic genes, including Bax, caspase-9, and caspase-3, in order to promote the activation of apoptotic signaling pathway [30]. In addition, Xinfeng Yu *et al*. revealed that suppression of miR-203 induced bcl-2 [31]. Hence, we suggest that CAV1 may regulate PI3K/AKT signaling pathway under the control of miR-203.

**Figure 11.**
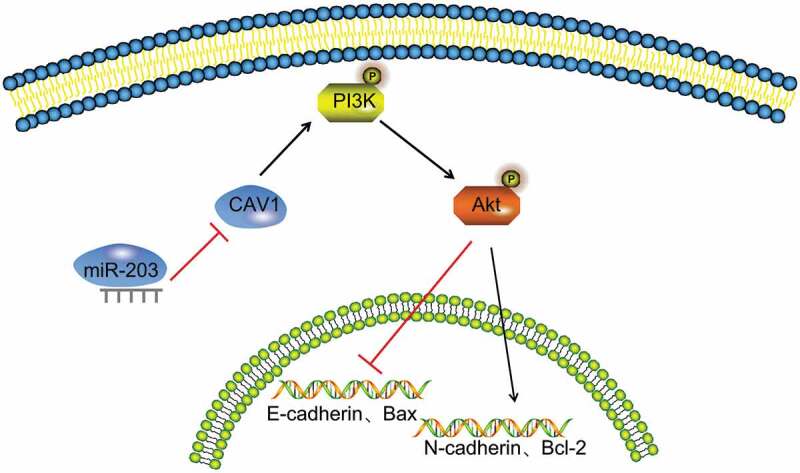
Mechanism of miR-203 targeting CAV1 and PI3K/AKT signaling pathway in RCC

Notes: miR-203 inhibits epithelial-mesenchymal transition, proliferation, migration and invasion, but promotes apoptosis of RCC cells via the inactivation of the PI3K/AKT signaling by inhibiting CAV1; miR-203, microRNA 203; CAV1, caveolin-1; PI3K, phosphoinositide 3-kinase; AKT, protein kinase B; bcl-2, B-cell lymphoma-2; Bax, bcl-2-Associated X.

In conclusion, our findings showed that miR-203 was down-regulated in RCC tissues. Furthermore, this study demonstrated that up-regulation of miR-203 inhibited cell proliferation, migration, invasion, and EMT by down-regulating CAV1 and inhibiting the PI3K/AKT signaling pathways in RCC, as confirmed by in vitro experiments (Figure 11). Collectively, our findings suggest that miR-203 serves as a potential therapeutic target of RCC. However, one miRNA may have multiple targets, and a target gene may correspond to different miRNAs. For instance, miR-203 can induce cell apoptosis by directly targeting Yes-1 [32] and inhibit EMT by downregulating Snai2 [33]. At present, there exist a few miRNAs with well-understood bio-functions, and the regulation of the downstream target genes by miRNAs is a very complicated reverse regulation network, which requires further studies.

## Materials and methods

### Ethical statement

This study protocol was approved by the Institutional Review Board of China-Japan Union Hospital of Jilin University. The acquisition of sample tissues was informed of patients, and written informed consent was obtained from all patients.

### Bioinformatics prediction

Original gene dataset data of RCC gene expression (GSE53757, GSE14762, GSE77199, and GSE6344) were downloaded from the Gene Expression Omnibus (GEO) (http://www.ncbi.nlm.nih.gov/geo) of the National Center for Biotechnology Information (NCBI) and the detailed information of gene dataset annotation platform and samples are shown in Table 3. The robust multiarray average (RMA) algorithm [34] of Affy installation package of R language [34] was employed for background correction, standardization, and other pretreatment of the expression data to filter false positive data. The Limma installation package of R language [35] was applied to select DEGs in RCC tissues and to generate heatmaps of DEGs, with *P.Value* < 0.05 and |LogFoldChange| > 2.0 used as screening values. The Venn online analysis tool-Calculate and draw custom Venn diagrams (http://bioinformatics.psb.ugent.be/webtools/Venn/) was employed to compare the DEGs from 4 gene datasets. DisGeNET database (http://www.disgenet.org/web/DisGeNET/menu/search?4), as a comprehensive platform that provides information about human disease-related genes and mutations [36], was used to retrieve genes related to kidney cancer with key words ‘RCC’. String database (http://www. string-db.org/), providing known or predicted protein-related information, was also employed to analyze the interaction between DEGs of RCC and disease genes and the enrichment of KEGG pathway. The confidence score was set to be greater than 0.4, and the Cytoscape 3.6.0 software [37] was used to visualize the interaction network of genes. The miRNAs that could regulate the DEGs were predicted using three miRNA-mRNA prediction tools, MicroRNA (http://34.236.212.39/microrna/getGeneForm.do), TargetScan (http://www.targetscan.org/vert_71/) and mirDIP (http://ophid.utoronto.ca/mirDIP/).

**Table 3.** RCC gene dataset (GSE53757, GSE14762, GSE77199 and GSE6344) information

Accession	Platform	Organism	Sample
GSE6344	GPL97	Homo sapiens	10 normal and 10 renal cell carcinoma tumors
GSE53757	GPL570	Homo sapiens	72 renal cell carcinoma tumor tissues and 72 normal kidneys
GSE14762	GPL6480	Homo sapiens	10 renal cell carcinoma tumor samples and 12 normal renal tissue
GSE77199	GPL97	Homo sapiens	4 healthy kidney endothelial sample and 4 renal cancer endothelial sample

Notes: RCC, renal cell carcinoma.

### Study subjects

Cancer and adjacent normal tissues were obtained from 112 patients who were pathologically diagnosed as RCC by experienced pathologist at China–Japan Union Hospital of Jilin University from April 2014 to August 2016. The patients enrolled included 68 males and 44 females, within the age of 24–68 years. The adjacent normal tissues located at 5 cm from the carcinomas were resected to serve as controls. None of the patients received anticancer therapies such as radiotherapy, chemotherapy, or immunotherapy prior to nephrectomy. Based on the clinicopathological staging of the Union for International Cancer Control/American Joint Committee on Cancer, the tumor tissues were classified as follows: 58 cases at stage I/II; 54 cases at stage III/IV. All tissue samples were preserved in liquid nitrogen or refrigerator at −80°C for further experiments.

### Immunohistochemistry (IHC)

Tissue samples were fixed with 10% formalin, embedded with paraffin, cut into 4 μm serial sections, and baked at 60°C for 1 h. Then, the sections were dewaxed with conventional xylene, dehydrated with gradient alcohol, and incubated in 3% H_2_O_2_ (Sigma-Aldrich Chemical Company, St Louis, MO, USA) at 37°C for 30 min. The sections were boiled in 0.01 M citric acid buffer at 95°C for 20 min, and cooled to room temperature. Subsequently, the sections were blocked with normal goat serum at 37°C for 10 min, added with primary mouse anti-human CAV1 antibody, and incubated at 4°C for 12 h. The sections were incubated with corresponding biotinylated secondary antibody of goat anti-mouse at room temperature for 10 min. Then, sections were added with streptavidin-horseradish peroxidase (S-A/HRP) to react at room temperature for 10 min, and colored by diaminobenzidine. After being placed in a darkroom at room temperature for 8 min, sections were counterstained with hematoxylin, dehydrated, cleared, mounted, and observed under a light microscope. Three different high-magnification fields (× 200) with the same size were randomly selected in each section, and the number of positive cells was counted in each section with the use of the Nikon image analysis software (NIS-Elements, Shanghai Henghao Instruments Co., Ltd, Shanghai, China).

### Reverse-transcription quantitative polymerase chain reaction (RT-qPCR)

Total RNA was extracted by StarPure RNA extraction Kit (No. D203-01, GenStar BioSolutions Co., Ltd., Beijing, China). Primers of miR-203, CAV1, PI3K, AKT, β-catenin, E-cadherin, N-cadherin, B-cell lymphoma-2 (bcl-2), bcl2-Associated X (Bax) and glyceraldehyde 3-phosphate dehydrogenase (GAPDH, Table 4) were designed and synthesized by TaKaRa Biotechnology Ltd., (Dalian, China). Following the instructions of TaqMan^TM^ MicroRNA Assays Reverse Transcription Kit (No.4366596, Thermo Fisher Scientific, Waltham, MA, USA), reverse transcription (20 μL) was reacted. Subsequently, real-time fluorescence quantitative PCR was carried out in a system ABI PRISM® 7300 (Prism® 7300, KunKe, Shanghai, China) in accordance with the instructions of the SYBR® Premix Ex Taq^TM^ II Kit (RR820A, Xingzhi Biological Technology, Guangzhou, China). U6 gene was taken as the internal control for miR-203, while GAPDH (No. abs830032, Absin Bioscience Inc., Shanghai, China) served as the internal control for CAV1, PI3K, AKT, β-catenin, E-cadherin, N-cadherin, bcl-2, and Bax. The 2^−ΔCt^ method represented the multiple proportions of the gene expression between the RCC group and the control group. These procedures could also be applied for RT-qPCR detection in cells.

**Table 4.** Primer sequences for reverse transcription quantitative polymerase chain reaction

Genes	Primer sequences
miR-203	F: 5ʹ-GGGTTGTGGAGGATTAGTT-3ʹ
	R: 5ʹ-AAACAACTAAACTCCAAACA-3’
U6	F: 5ʹ-CGCTTCGGCAGCACATATACTAA-3ʹ
	R: 5ʹ-TATGGAACGCTTCACGAATTTGC-3’
CAV1	F: 5ʹ-TGACTGAGAAGCAAGTGTATGACG-3ʹ
	R: 5ʹ-GCAGAAGGTATGGACGTAGAT-3’
PI3K	F: 5ʹ-CATCACTTCCTCCTGCTCTAT-3ʹ
	R: 5ʹ-CAGTTGTTGTCAATCTTCTTC-3’
AKT	F: 5ʹ-ACGATGAATGAGGTGTCTGT-3ʹ
	R: 5ʹ-TCTGCTACGGTGAAGTTGTT-3’
β-catenin	F: 5ʹ-GCTGATTTGATGGAGTTGGA-3ʹ
	R: 5ʹ-TCAGCTACTTGTTCTTGAGTGAA-3’
E-cadherin	F: 5ʹ-AACATGGTTCAGATCAAATC-3ʹ
	R: 5ʹ-AAGCTTGAAGATCGGAGGATTATCG-3’
N-cadherin	F: 5ʹ-CAACTTGCCAGAA AACTCCAGG-3ʹ
	R: 5ʹ-ATGAAACCGGGCTATCTGCTC-3’
bcl-2	F: 5ʹ-TATAAGCTGTCGCAGAGGGG-3ʹ
	R: 5ʹ-TGACGCTCTCCACACACATG-3’
Bax	F: 5ʹ-TGCCAGCAAACTGGTGCTCA-3ʹ
	R: 5ʹ-GCACTCCCGCCACAAAGATG-3’
GAPDH	F: 5ʹ-GGGCTGCTTTTAACTCTGGT-3ʹ
	R: 5ʹ-GCAGGTTTTTCTAGACGG-3’

Notes: miR-203, microRNA 203; CAV1, caveolin-1; PI3K, phosphoinositide 3-kinase; AKT, protein kinase B; bcl-2, B-cell lymphoma-2; Bax, bcl-2-Associated X; GAPDH, glyceraldehyde-3-phosphate dehydroge

### Western blot analysis

Extracted sample tissues were added with liquid nitrogen, grinded to a fine powder with a uniform appearance and then added with 1 mL histiocyte lysate composed of 50 mmol/L Tris, 150 mmol/L NaCl, 5 mmol/L ethylene diamine tetraacetic acid (EDTA), 0.1% sodium dodecyl sulfate (SDS), 1% NP-40, 5 μg/mL Aprotinin and 2 mmol/L phenylmethanesulfonyl fluoride. After being homogenated in ice bath, tissues were cracked with an addition of protein lysis buffer at 4°C for 30 min with repeated shaking every 10 min, followed by 20-min centrifugation at 4°C at 12,000 rpm with the lipid layer was discarded. The supernatant was collected to detect protein concentration using the bicinchoninic acid Protein Quantification Kit (No.20201ES76, Yeasen Group Limited, Shanghai, China). A total of 30 μg protein was collected to load samples, and the protein concentration was adjusted by deionized water. After the preparation of 10% SDS stacking gel and separating gel, samples were mixed with the loading buffer and boiled for 5 min at 100°C. Following ice bath and centrifugation, the mixture was added to each lane at equal volume with a micro sampler, followed by electrophoresis. Subsequently, the proteins on the gel were transferred to a nitrocellulose membrane. The membrane was blocked with 5% skim milk powder overnight at 4°C, and incubation was carried out with the following primary rabbit anti-human antibodies (Abcam, Cambridge, UK) to CAV1 (1: 1000, ab32577), β-catenin (1: 5000, ab32572), bcl-2 (1: 1000, ab32124), Bax (1: 2000, ab32503), PI3K (1: 1000, ab151549), AKT (1: 500, ab8805), phosphorylated AKT (1: 1000, ab38449), E-cadherin (1: 10,000, ab40772), and N-cadherin (1: 1000, ab76057) overnight at 4°C. Afterward, the membranes were immersed in the enhanced chemiluminescence reagent (Pierce, Waltham, MA, USA) for 1 min at room temperature with liquid removed, and covered with plastic-wrap. The membrane was exposed by Ray, developed and fixed under dark conditions for blot observation. GAPDH served as internal control, and the ratio of the gray value of the target band to the internal reference band was regarded as the relative expression of the protein. This method can also be applied when conducting western blot analysis in cells.

### Dual-luciferase reporter gene assay

The bioinformatics prediction website (microRNA.org) was used for the target gene analysis of miR-203, and dual-luciferase reporter assay was performed to confirm whether CAV1 is the direct target gene of miR-203. The full-length 3ʹUTR region of CAV1 gene was cloned and amplified. After being digested using restriction enzyme Xba I and Xho I, the target fragments were attached to pmirGLO Dual-Luciferase miRNA Target Expression Vector (No. E1330, Promega, Madison, Wisconsin, USA) and named p CAV1-wild type (wt). Next, bioinformatics software was used to predict the site of miR-203 binding to the target gene CAV1 to locate the mutation. The p CAV1-mutant type (mut) vector was constructed, and the Renilla luciferase-expressing vector pRL-TK (No. E2241, Promega) was used as internal control. The miR-203 mimic and miR-203 NC were co-transfected with luciferase reporter vectors respectively into HEK-293 T cells, followed by the detection of fluorescence intensity using GloMax® 20/20 Luminometer (No.E5311, Promega) and the analysis of data obtained during this procedure.

### Cell line screening

Human renal clear cell carcinoma (RCCC) cells (786-O, ACHN, OS-RC-2 and ketr-3) were purchased form Cell Resource Center, Shanghai Institutes for Biological Sciences, Chinese Academy of Sciences (Shanghai, China). The cells were cultured in Roswell Park Memorial Institute (RPMI) 1640 medium (No.11875093, Thermo Fisher Scientific) containing 10% fetal bovine serum (FBS) in a humidified incubator with 5% CO_2_ at 37°C, and subcultured every 2 to 3 days. Finally, RT-qPCR and western blot analysis were conducted for the detection of mRNA and protein levels of CAV1, and cells with the highest expression of CAV1 were selected for subsequent experiments.

### Cell grouping and transfection

Selected cancer cells were classified into the following groups: blank (transfection without any sequence) group, NC group (transfection with NC sequence), miR-203 mimic group (transfection with miR-203 mimic), miR-203 inhibitor group (transfection with miR-203 inhibitor), siRNA-CAV1 group (transfection with siRNA-CAV1) and miR-203 mimic + siRNA-CAV1 group (transfection with miR-203 mimic and siRNA- CAV1). Cells were seeded in a 6-well plate at 24 h prior to transfection and then transfected in accordance with the instructions of Lipofectamin 2000 (No. 11,668–019, Invitrogen Inc., Carlsbad, CA, USA) when cell confluence reached 30–50%. Cells were incubated at 37°C with 5% CO_2_ for 6 to 8 h. The medium was replaced with complete medium, and cells were cultured for additional 24 to 48 h for follow-up experiments.

### 3-(4, 5-dimethylthiazol-2-yl)-2, 5-diphenyltetrazolium bromide (MTT) assay

After 48 h of transfection, the cells were harvested for counting. The cells were seeded in a 96-well plate at a density of 3 × 10^3^ to 6 × 10^3^ cells per well with a volume of 100 μL set in each well, and cultured. A total of 6 duplicated wells were set. The following experiments were carried out at 24 h, 48 h, and 72 h after the incubation: each well was added with 20 μL MTT solution (5 mg/mL), and incubated at 37°C for 4 h. Afterward, the incubation was terminated and the culture supernatant fluid was discarded. Next, each well was added with 150 μL dimethyl sulfoxide and the optical density (OD) of each well at 570 nm was measured by enzyme-linked immunometric meter (NYW-96 M, Beijing Noyawei Instrument Co., Ltd., Beijing, China). The cell viability curve was drawn with time point as its abscissa and OD value as the ordinate.

### Immunofluorescence

Following 48-h cell transfection, the cells were seeded on slides in a 6-well plate with 1 × 10^5^ cells per well. After 48 h of culture, the expression of E-cadherin and N-cadherin was detected by immunofluorescence staining. The slides were fixed with pre-cooled methanol for 10 min, treated with 0.5% TritoneX 100 for 15 min at room temperature, and then sealed with 30 mL/L normal goat serum working solution for 30 min. Following the removal of the blocking solution, the cells were added with mouse anti-human primary antibody (1: 1000) at 4°C overnight. Then, the secondary goat anti-mouse immunoglobulin G antibody (1: 500) was added into cells which were incubated at 37°C for 30 min. Cells were cultured at room temperature for 30 min with 200 μg/mL RNase, and counterstained with 5 μmol/L nucleic acid fluorescent dye TOPRO3 for 5 min. The slides were dried naturally and sealed with buffer glycerin. The sections were observed and scanned under a LeicaTCSSP laser confocal microscope. PBS was used as a NC.

### Scratch test

First, cells were seeded into a 6-well plate, and following the adherence of the cells to the wall, the culture medium was replaced by serum-free Dulbecco’s modified eagle medium. When the cell confluence reached 90–100%, a sterile 10 μL pipette tip was used to slowly scratch the bottom of the 6-well plate in a vertical position, leaving approximately 4 to 5 wounds with the same width on each well. Then, the cells were rinsed 3 times with phosphate buffer saline (PBS) to remove cellular debris, and placed in an incubator. Cell migration distances in the wound were observed using an inverted microscope at 0 h and 24 h after the scratching. Multiple fields were randomly selected and photographed. Each experiment group was set with 3 duplicated wells to warrant the credibility and accuracy of experimental results.

### Transwell assay

The Transwell chamber was placed in a 24-well plate. The apical chamber of the basement membrane was coated with Matrigel (diluted 1: 8), and dried at room temperature. After routine digestion, cells were resuspended with RPMI 1640 medium (No.11875093, Thermo Fisher Scientific) with cell density adjusted to 1 × 10^5^ cells/mL. Then, 200 μL cell suspension was added into the apical chamber of the Transwell chamber covered with Matrigel, and 600 μL RPMI 1640 medium containing 20% FBS was added to the basolateral chamber. After conventional culture for 24 h, cells in the apical chamber of the Transwell chamber was wiped with a cotton swab. Subsequently, cells were fixed with 4% paraformaldehyde for 15 min, and stained with 0.5% crystal violet solution (formulated with methanol) for 15 min. Five fields (200 ×) were randomly selected and photographed using an inverted microscope (No.XDS-800D, Shanghai Caikang Optics Instrument Co., Ltd., Shanghai, China). The transmembrane cells were counted. Each experiment group was set with three duplicated wells.

### Flow cytometry

After 48 h of transfection, the cells underwent centrifugation with the removal of supernatant, and re-suspension was carried out with PBS with cell concentration adjusted to about 1 × 10^5^ cells/mL. Cells were fixed at 4°C with 1 mL 75% ethanol precooled at −20°C for 1 h, centrifuged with removal of cool ethanol, and washed twice with PBS to remove the supernatant. Next, the cells were added with 100 μL ribonuclease A (RNase) under dark conditions, water-bathed at 37°C for 30 min, stained, and completely mixed with 400 μL propidium iodide (PI) (No. P4170, Sigma-Aldrich Chemical Company), and then placed in the dark at 4°C for 30 min. Afterward, a flow cytometer was employed to record the red fluorescence at excitation wavelength of 488 nm to detect cell cycle.

After 48 h of transfection, the cells were detached from the culture surface with trypsin containing no EDTA, collected into a flow tube, and centrifuged. After the removal of supernatant, further centrifugation was carried out with supernatant discarded. Next, Annexin-V-fluorescein isothiocyanate (FITC)/PI dye was prepared with Annexin-V-FITC, PI, and 4-(2-hydroxyethyl)-1-piperazineëthanesulfonic acid (HEPES) at ratio of 1: 2: 50 in accordance with the instructions of Annexin-V-FITC apoptosis detection kit (Sigma-Aldrich Chemical Company). Cells (1 × 10^6^) were re-suspended with 100 μL of dye liquor, mixed by shaking, incubated at room temperature for 15 min, added with 1 mL HEPES, and mixed. The FITC and PI fluorescences were detected at the excitation wavelength of 485 nm, 525 nm, and 620 nm bandpass filters, respectively, for the detection of cell apoptosis.

### Statistical analysis

All data were processed using SPSS 21.0 statistical software (IBM Corp., Armonk, NY, USA) and expressed as mean ± standard deviation. The *t*-test was used to compare two groups while one-way analysis of variance was used for multiple group comparison. Pairwise comparison among multiple groups was analyzed using Tukey’s posttest, and data at different time points were compared with repeated measurement analysis of variance. *p* < 0.05 indicated a statistically significant value.

## References

[1] Li Y, Ding YU, Chen D, et al. Renal cell carcinoma growing into the renal pelvis and mimicking transitional cell carcinoma: a case report and literature review. Oncol Lett. 2015;9(4):1869–1872. 25789058 10.3892/ol.2015.2898 PMC4356286

[2] Ljungberg B, Campbell SC, Choi HY, et al. The epidemiology of renal cell carcinoma. Eur Urol. 2011;60(4):615–621. 21741761 10.1016/j.eururo.2011.06.049

[3] Yu CP, Ho JY, Huang YT, et al. Estrogen inhibits renal cell carcinoma cell progression through estrogen receptor-beta activation. PLoS One. 2013;8(2):e56667. 23460808 10.1371/journal.pone.0056667 PMC3584057

[4] Chow WH, Devesa SS. Contemporary epidemiology of renal cell cancer. Cancer J. 2008;14(5):288–301. 18836333 10.1097/PPO.0b013e3181867628 PMC3077538

[5] Medina-Rico M, Ramos HL, Lobo M, et al. Epidemiology of renal cancer in developing countries: review of the literature. Can Urol Assoc J. 2018;12(3):E154–E162. 29283089 10.5489/cuaj.4464 PMC5869042

[6] Labochka D, Moszczuk B, Kukwa W, et al. Mechanisms through which diabetes mellitus influences renal cell carcinoma development and treatment: A review of the literature. Int J Mol Med. 2016;38(6):1887–1894. 27748835 10.3892/ijmm.2016.2776

[7] Peng J, Mo R, Ma J, et al. let-7b and let-7c are determinants of intrinsic chemoresistance in renal cell carcinoma. World J Surg Oncol. 2015;13(1):175. 25951903 10.1186/s12957-015-0596-4 PMC4426556

[8] Stewart GD, O’Mahony FC, Powles T, et al. What can molecular pathology contribute to the management of renal cell carcinoma? Nat Rev Urol. 2011;8(5):255–265. 21487387 10.1038/nrurol.2011.43

[9] Li W, Liu M, Feng Y, et al. Downregulated miR-646 in clear cell renal carcinoma correlated with tumour metastasis by targeting the nin one binding protein (NOB1). Br J Cancer. 2014;111(6):1188–1200. 25010867 10.1038/bjc.2014.382 PMC4453839

[10] Su Z, Chen D, Li Y, et al. microRNA-184 functions as tumor suppressor in renal cell carcinoma. Exp Ther Med. 2015;9(3):961–966. 25667660 10.3892/etm.2015.2199 PMC4316952

[11] Xiang C, Cui SP, Ke Y. MiR- 144 inhibits cell proliferation of renal cell carcinoma by targeting MTOR. J Huazhong Univ Sci Technolog Med Sci. 2016;36(2):186–192. 27072960 10.1007/s11596-016-1564-0

[12] Chim CS, Wong KY, Leung CY, et al. Epigenetic inactivation of the hsa-miR-203 in haematological malignancies. J Cell Mol Med. 2011;15(12):2760–2767. 21323860 10.1111/j.1582-4934.2011.01274.x PMC4373446

[13] Xu M, Gu M, Zhang K, et al. miR-203 inhibition of renal cancer cell proliferation, migration and invasion by targeting of FGF2. Diagn Pathol. 2015;10(1):24. 25890121 10.1186/s13000-015-0255-7 PMC4419389

[14] Campbell L, Al-Jayyoussi G, Gutteridge R, et al. Caveolin-1 in renal cell carcinoma promotes tumour cell invasion, and in co-operation with pERK predicts metastases in patients with clinically confined disease. J Transl Med. 2013;11(1):255. 24119769 10.1186/1479-5876-11-255 PMC4015803

[15] Chow TF, Youssef YM, Lianidou E, et al. Differential expression profiling of microRNAs and their potential involvement in renal cell carcinoma pathogenesis. Clin Biochem. 2010;43(1–2):150–158. 19646430 10.1016/j.clinbiochem.2009.07.020

[16] Ciccarese C, Brunelli M, Montironi R, et al. The prospect of precision therapy for renal cell carcinoma. Cancer Treat Rev. 2016;49:37–44. 27453294 10.1016/j.ctrv.2016.07.003

[17] Betel D, Koppal A, Agius P, et al. Comprehensive modeling of microRNA targets predicts functional non-conserved and non-canonical sites. Genome Biol. 2010;11(8):R90. 20799968 10.1186/gb-2010-11-8-r90 PMC2945792

[18] Zhou K, Liu M, Cao Y. New insight into microRNA functions in cancer: oncogene-microRNA-tumor suppressor gene network. Front Mol Biosci. 2017;4: 46. 28736730 10.3389/fmolb.2017.00046 PMC5500619

[19] Wu D, Pan H, Zhou Y, et al. microRNA-133b downregulation and inhibition of cell proliferation, migration and invasion by targeting matrix metallopeptidase-9 in renal cell carcinoma. Mol Med Rep. 2014;9(6):2491–2498. 24714873 10.3892/mmr.2014.2116

[20] Zhang Z, Zhang B, Li W, et al. Epigenetic Silencing of miR-203 upregulates SNAI2 and contributes to the invasiveness of malignant breast cancer cells. Genes Cancer. 2011;2(8):782–791. 22393463 10.1177/1947601911429743 PMC3278899

[21] Zhu Y, Xu L, Zhang J, et al. Klotho suppresses tumor progression via inhibiting PI3K/Akt/GSK3beta/Snail signaling in renal cell carcinoma. Cancer Sci. 2013;104(6):663–671. 23433103 10.1111/cas.12134 PMC7657207

[22] Lin J, Lin Y, Fan L, et al. miR-203 inhibits cell proliferation and promotes cisplatin induced cell death in tongue squamous cancer. Biochem Biophys Res Commun. 2016;473(2):382–387. 26946357 10.1016/j.bbrc.2016.02.105

[23] Yang H, Guan L, Li S, et al. Mechanosensitive caveolin-1 activation-induced PI3K/Akt/mTOR signaling pathway promotes breast cancer motility, invadopodia formation and metastasis in vivo. Oncotarget. 2016;7(13):16227–16247. 26919102 10.18632/oncotarget.7583 PMC4941310

[24] Liang W, Hao Z, Han JL, et al. CAV-1 contributes to bladder cancer progression by inducing epithelial-to-mesenchymal transition. Urol Oncol. 2014;32(6):855–863. 24968949 10.1016/j.urolonc.2014.01.005

[25] Butz H, Szabo PM, Khella HW, et al. miRNA-target network reveals miR-124as a key miRNA contributing to clear cell renal cell carcinoma aggressive behaviour by targeting CAV1 and FLOT1. Oncotarget. 2015;6(14):12543–12557. 26002553 10.18632/oncotarget.3815 PMC4494957

[26] Hall DP, Cost NG, Hegde S, et al. TRPM3 and miR-204 establish a regulatory circuit that controls oncogenic autophagy in clear cell renal cell carcinoma. Cancer Cell. 2014;26(5):738–753. 25517751 10.1016/j.ccell.2014.09.015 PMC4269832

[27] Han F, Zhang L, Zhou Y, et al. Caveolin-1 regulates cell apoptosis and invasion ability in paclitaxel-induced multidrug-resistant A549 lung cancer cells. Int J Clin Exp Pathol. 2015;8(8):8937–8947. 26464635 PMC4583867

[28] Xiong J, Wang D, Wei A, et al. Deregulated expression of miR-107 inhibits metastasis of PDAC through inhibition PI3K/Akt signaling via caveolin-1 and PTEN. Exp Cell Res. 2017;361(2):316–323. 29111166 10.1016/j.yexcr.2017.10.033

[29] Zhao Z, Liu H, Hou J, et al. Tumor protein D52 (TPD52) inhibits growth and metastasis in renal cell carcinoma cells through the PI3K/Akt signaling pathway. Oncol Res. 2017;25(5):773–779. 27983909 10.3727/096504016X14774889687280 PMC7841249

[30] Lim HS, Kim CS, Kim JS, et al. Suppression of oral carcinoma oncogenic activity by microRNA-203 via down-regulation of SEMA6A. Anticancer Res. 2017;37(10):5425–5433. 28982852 10.21873/anticanres.11970

[31] Yu X, Zhang X, Dhakal IB, et al. Induction of cell proliferation and survival genes by estradiol-repressed microRNAs in breast cancer cells. BMC Cancer. 2012;12(1):29. 22260523 10.1186/1471-2407-12-29 PMC3274428

[32] Lee SA, Kim JS, Park SY, et al. miR-203 downregulates Yes-1 and suppresses oncogenic activity in human oral cancer cells. J Biosci Bioeng. 2015;120(4):351–358. 25910964 10.1016/j.jbiosc.2015.02.002

[33] Zhao G, Guo Y, Chen Z, et al. miR-203 functions as a tumor suppressor by inhibiting epithelial to mesenchymal transition in ovarian cancer. J Cancer Sci Ther. 2015;7(2):34–43. 26819680 10.4172/1948-5956.1000322 PMC4725318

[34] Irizarry RA, Hobbs B, Collin F, et al. Exploration, normalization, and summaries of high density oligonucleotide array probe level data. Biostatistics. 2003;4(2):249–264. 12925520 10.1093/biostatistics/4.2.249

[35] Smyth GK. Linear models and empirical bayes methods for assessing differential expression in microarray experiments. Stat Appl Genet Mol Biol. 2004;3(1):Article3. 16646809 10.2202/1544-6115.1027

[36] Pinero J, Bravo A, Queralt-Rosinach N, et al. DisGeNET: a comprehensive platform integrating information on human disease-associated genes and variants. Nucleic Acids Res. 2017;45(D1):D833–D839. 27924018 10.1093/nar/gkw943 PMC5210640

[37] Shannon P, Markiel A, Ozier O, et al. Cytoscape: a software environment for integrated models of biomolecular interaction networks. Genome Res. 2003;13(11):2498–2504. 14597658 10.1101/gr.1239303 PMC403769

